# Effect of Adding Silica Nanoparticles on the Physicochemical Properties, Antimicrobial Action, and the Hardness of Dental Stone Type 4

**DOI:** 10.1155/2022/4762017

**Published:** 2022-04-26

**Authors:** Navid Aghbolaghi, Solmaz Maleki Dizaj, Ramin Negahdari, Amir Reza Jamei Khosroshahi, Yashar Rezaei, Sepideh Bohlouli, Mohammad Ali Ghavimi

**Affiliations:** ^1^Department of Prosthodontics, Faculty of Dentistry, Tabriz University of Medical Sciences, Tabriz, Iran; ^2^Dental and Periodontal Research Center, Tabriz University of Medical Sciences, Tabriz, Iran; ^3^Department of Dental Biomaterials, Faculty of Dentistry, Tabriz University of Medical Sciences, Tabriz, Iran; ^4^Department of Pediatric Dentistry, Faculty of Dentistry, Tabriz University of Medical Sciences, Tabriz, Iran; ^5^Department of Oral Medicine, Faculty of Dentistry, Tabriz University of Medical Sciences, Tabriz, Iran; ^6^Department of Oral and Maxillofacial Surgery, Faculty of Dentistry, Tabriz University of Medical Sciences, Tabriz, Iran

## Abstract

This study was conducted to investigate the effect of adding silica nanoparticles on the physicochemical properties, antimicrobial action, and the hardness of dental stone type 4. Dental stone type 4 powder was physically mixed with nanoparticle powder at weight percentages (0, 0.5, 1, and 2 percent). The required amount of powder was added to water according to the manufacturer's instructions. The prepared set materials were subjected to the physicochemical studies; Fourier transmission infrared spectroscopy (FTIR) was taken up to investigate the functional groups and X-ray diffraction (XRD) was used to evaluate the crystallinity. Also, scanning electron microscopy (SEM) was used to examine the morphology of the prepared samples. Agar diffusion test was carried out for the prepared samples against the *Escherichia coli* (*E. coli*) and *Staphylococcus aureus* (*S. aureus*) to test the average growth inhibition zones. Finally, the Vickers surface hardness test was performed for each group using a hardness tester. The adding silica nanoparticles to dental stone type 4 increased the diameter of inhibition zones for the groups in both bacteria significantly (*p* < 0.05). The results showed that adding silica nanoparticles to dental stone type 4 increased the diameter of inhibition zones for the groups in both bacteria significantly (*p* < 0.0001). There was a significant difference between all groups and the 0% group in both bacteria (*p* < 0.0001). Besides, the adding of silica nanoparticles to dental stone type 4 increased the surface hardness significantly (*p* = 0.0057) without any effect on physicochemical properties. The 0% and the 0.5% groups had significant differences with the 2% group (*p* = 0.0046 and *p* = 0.0205 respectively). Then, at least 2% silica nanoparticles are needed for a significant increase. Clinical trials are needed to enlarge for dental stone type 4 containing silica nanoparticles in the future.

## 1. Introduction

Gypsum, known also as calcium sulfate dihydrate (CaSO_4_.2H_2_O), is a mineral material that originates in nature as compressed or twinned crystals and clear cleavable masses recognized as selenite. Gypsum is also accessible in dense and grainy arrangements. This material is extensively utilized for the production of dental casts and laboratory processes [1].

In dentistry, abrasion resistance and cast hardness are of serious significance for working casts and dies as they undergo laboratory processes such as wax-up, fit checking of castings, and ultimate polishing.

Some reports have shown that the assessment of the physicochemical properties is necessary to explain the adsorption, coagulation, stability, flotation, and viscosity of dental materials such as cements and stones [2]. Microbial contamination of dental stones is also another main factor that should be considered. Microorganisms may originate in stones from contaminated impressions, turning dental casts into a potential font of cross-contamination [3, 4]. Dental stones can also be reinfected during manufacturing of a prosthesis [5]. Hardness is another main property for a dental stone material and is defined as the resistance to indentation. It can be measured by determining the perpetual depth of the indentation [6].

Based on the reports, various treatments have been suggested to enhance the physicochemical, mechanical, and antimicrobial action of dental stones. Inorganic filler particles can be applied for dental materials to improve their properties. Some particles such as quartz, colloidal silica, strontium, silica glass comprising barium, and zirconia have been applied in dental materials as diverse kinds of inorganic fillers [7]. A recent novelty in inorganic fillers has been the utilization of nanotechnology, with the main goal of improving their properties [8].

Nanotechnology is the science of the manufacturing and manipulation of materials in the range of 100 nm by various methodologies [9, 10]. The appearance of nanomaterials, including adhesives and composite resins, has been modified by these technological progressions [11, 12]. The novel and modified nanomaterials have improved the mechanical, antimicrobial, and physical properties of materials resulting in better clinical enactment [13]. Silica-based nanoparticles have shown an important part in nanotechnology, owing to their size, surface area, biocompatibility, antimicrobial action, low toxicity, low density, and high adsorption capacity [14].

Hence, this study was undertaken to test the impact of adding silica nanoparticles on the physicochemical properties, morphology, the antimicrobial action, and the hardness of dental stone type 4.

## 2. Materials and Methods

### 2.1. Materials

Silica nanoparticles with an average particle size of 50 nm were purchased from Tamad Kala Company (Iran-Tehran). Dental stone type 4 (SNOW ROCK) with 10 minutes setting time was purchased from Dk Mungyo Corporation- South Korea.

### 2.2. Sample Preparation

In this study, the specimens were divided into four groups. In test group 1, 0.5 percent silica nanoparticles were added to dental stone, while test groups 2 and 3 had 1% and 2% nanoparticles, respectively. Dental stone without the addition of silica nanoparticles was used as a control group. Dental stone type 4 powder was physically mixed with a nanoparticle powder at weight percentages of 0.5, 1, and 2%. The mixture was sonicated for one hour to prevent powder agglomeration. The required amount of powder was added to the water according to the manufacturer's instructions (powder/water ratio of 100 g/0.24 mL, setting time of 10 minute, and the temperature of 25 C°) and mixed until a uniform consistency was obtained (mixing time of 5 minute). For FTIR, XRD, and SEM tests, 100 *µ*g of samples were used for analyses. For the microbial test, the discs with a diameter of 6 mm were prepared from the obtained mix materials. Three disks were prepared for each specimen. For the hardness test, three disk were prepared (40 mm in diameter) for each specimen using plastic molds.

## 3. Characterization

The prepared samples were subjected to physicochemical studies as well as SEM images.

### 3.1. Physicochemical Studies

#### 3.1.1. X-Ray Diffraction (XRD)

The evaluation of crystallinity pattern (crystalline or amorphous state) of the die stone samples with and without silica nanoparticles XRD patterns was done at room temperature for set samples. The samples were exposed to an X-ray diffraction device (Siemens Germany, Model D5000) and irradiated with a wavelength of 1.5405 Å, a voltage of 40 kV of voltage, and a current of 30 mA, and their patterns were recorded by the device. X-ray diffraction (Siemens, Model D5000, Germany) was used to evaluate characteristics of crystallinity.

#### 3.1.2. Fourier Transmission Infrared Spectroscopy (FTIR)

Fourier transmission infrared spectroscopy (FTIR) was used to investigate possible connections and identification of functional groups. FTIR patterns were determined at room temperature for the set samples. For this purpose, 0.5 micrograms of the prepared samples were placed in the pan of the FTIR device (Shimadzu, Japan) and the device was adjusted in the wavelengths of 400 to 4000 (cm^−1^).

#### 3.1.3. Morphological Characterization

The evaluation of morphology and the mixing state of die stone samples with and without silica nanoparticles was performed using SEM. For this test, 2 *µ*g of samples were placed on a scanning electron microscope (SEM) plate and they were covered with a thin layer of gold (about 10 nm). The working distance was set at 10.19 mm and device voltage was set at 15 Kv. After setting the device and selecting the magnification (2.5 kx), the image was taken from the appropriate area on the screen under the microscope. Scanning electron microscopy (SEM, TESCAN, Warrendale, PA) was used to examine the size and the morphology of the prepared samples.

#### 3.1.4. Antimicrobial Test

*Staphylococcus aureus* (ATCC: 6538) and *Escherichia coli* (ATCC: 25922) were provided from the Pasteur Institute of Iran (Tehran, Iran). A disk diffusion technique was utilized to test the antimicrobial performance for the groups. Discs with a diameter of 6 mm were prepared from the obtained set materials. Then, 12 obtained set disks were classified into four groups (3 in each group). The first group contained 0.5% nanoparticles, the second group contained 1% nanoparticles, and the third group contained 2% nanoparticles. The fourth group without nanoparticles was considered for comparison (0% group as a negative control group). To compare the inhibition zone, vancomycin (30 mg per disc) and rifampicin (5 mg per disc) were employed (positive controls). Using microbiological plates, the culture medium of the Müller Hinton agar was made. Sterilized swabs were submerged in a microbial solution at a concentration of half McFarland (1.5 × 108), and subsequently, at 60°, lawn cultivation was conducted three times on the plate. After that, the swab was turned toward the middle part of the plate. After 24 hours of incubation at 35°C, the created disks of samples were put on the culture medium. The plates were evaluated for the diameter of the inhibition zone surrounding the disks after 24 hours of incubation.

#### 3.1.5. Vickers Surface Hardness

Three disk were prepared (40 mm in diameter) for each specimen using plastic molds. Then, the specimens with bubbles and cracks were excluded from the study. Then, 12 obtained set disks were classified into four groups (3 in each group). The first group contained 0.5% nanoparticles, the second group contained 1% nanoparticles, and the third group contained 2% nanoparticles. The fourth group without nanoparticles was considered for comparison. For each group, the Vickers surface hardness test was performed after 24 hours of setting in an incubator at 37°C.

The Vickers surface hardness test was performed using a hardness tester (HV-1000Z, PACE Technologies), equipped with a diamond indenter. Three indentations were obtained for each specimen using 0.5 N for 15 seconds and the average wickers hardness number of the three readings for each specimen was recorded [15]. The obtained data were subjected to statistical analysis.

### 3.2. Statistical Analysis

The results were reported as mean ± SD. The Shapiro–Wilk test was performed to determine whether the findings were normal. To compare the findings of among the investigated groups (both microbial and hardness tests), a one-way analysis of variance was employed due to normal data distribution. Tukey's post hoc test was used for the analysis between the groups. SPSS software (version 16, IBM, New York, USA) was used to analyze the data. The *p*-values of less than 0.05 were considered as the significance level.

## 4. Results and Discussion

**Figure 1:**
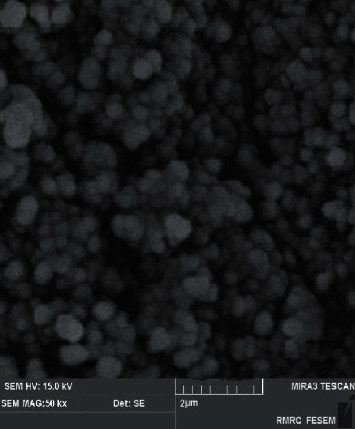
Silica nanoparticles with an average particle size of 50 nm.

**Figure 2:**
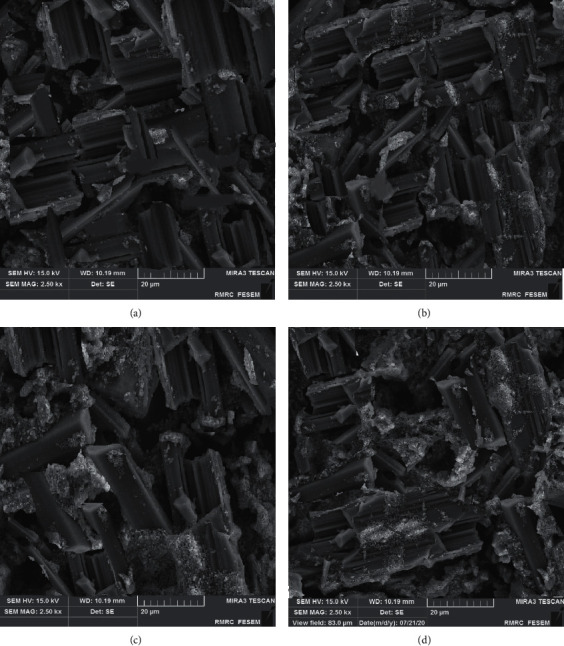
(a) SEM image for stone without silica nanoparticles, (b) stone containing 0.5% silica nanoparticles, (c) stone containing 1% silica nanoparticles, and (d) stone containing 2% silica nanoparticles.

Figure 1 shows silica nanoparticles with an average particle size of 50 nm and spherical morphology.  Figure 2 shows the SEM image for the stone containing silica nanoparticles; for stone without silica nanoparticles (a), stone containing 0.5% silica nanoparticles (b), stone containing 1% silica nanoparticles (c), and stone containing 2% silica nanoparticles (d).  Figure 2(a) shows the stone without silica nanoparticles, while the presence of nanoparticles in Figures 2(b)–2(d) can be observed clearly. The nanoparticles are distributed relatively homogeneous throughout the surface of the stone matrix. As it is clear, the stone particles are in micrometer size and the silica particles are in nanometer size. Micro-circular crystals of calcium sulfate can be observed for stone particles predominantly. There are some fine particles for dental stone as well related to the other component of stone (can be seen in Figure 2(a)). Suryawanshi et al. reported a similar morphology for calcium sulfate stones and the reason for the existing morphology was the presence of multidimensional sloping micro-circular crystals of calcium sulfate [16]. SEM results showed that after the addition of nanoparticles, there were no significant morphological changes in stone compared to the control group (stone without nanoparticles). Salah et al. reported similar results regarding the addition of silver nanoparticles to dental stone type 4. In their study, the addition of silver nanoparticles did not show any effect on the morphology of dental stone compared to the control without nanoparticles [17].

**Figure 3:**
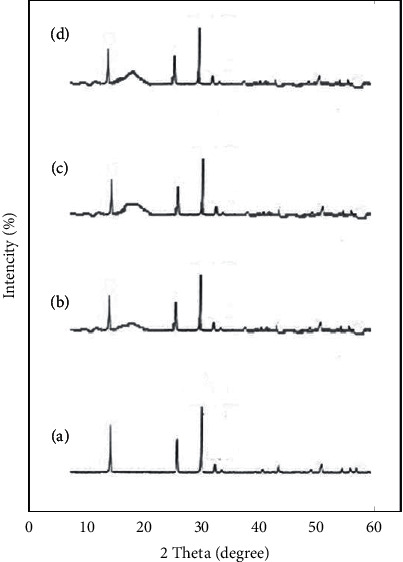
(a) Results for XRD for stone without silica nanoparticles, (b) stone containing 0.5% silica nanoparticles, (c) stone containing 1% silica nanoparticles, and (d) stone containing 2% silica nanoparticles.

Figure 3(a)–3(d) shows the results for the XRD test for the prepared materials. A wide low-intensity XRD peak at 20° in the stone containing 0.5% silica nanoparticles, the stone containing 1% silica nanoparticles, and the stone containing 2% silica nanoparticles is related to the presence of silica nanoparticles in the stone matrix. The comparison of XRD peaks with reference peaks in the sources also showed that the bronchitis polymorph of calcium phosphate is the most abundant polymorph in stone. Suriavanshi et al. reported a similar case of calcium phosphate stone [16]. Therefore, in this study, mixing nanoparticles with stone had no effect on its crystallinity (change of crystalline state to amorphous or polymorphic type).

**Figure 4:**
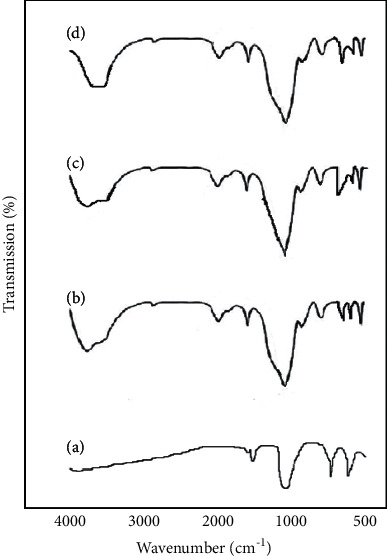
(a) Results for FTIR for stone without silica nanoparticles, (b) stone containing 0.5% silica nanoparticles, (c) stone containing 1% silica nanoparticles, and (d) stone containing 2% silica nanoparticles.

The results of the FTIR test for the prepared samples are presented in Figures 4(a)–4(d)). FTIR analysis displayed absorption as the main peaks for all functional groups of dental stone and silica nanoparticles. No additional peaks were observed in the whole of the area. The peaks at 1215 cm^−1^, 1156 cm^−1^, and 1062 cm^−1^ belong to the dual-band of P=O (from phosphate groups). The dual peak in the range of 3542–3500 cm^−1^ is related to the tensile peak of the OH group, which can indicate the adsorption of water by stone as well as silica nanoparticles [18].

### 4.1. Microbial Findings

Table 1 illustrates the inhibition zone measurements for the groups and the controls against *S. aureus*, and *E. coli*. The one-way ANOVA results showed that adding silica nanoparticles to dental stone type 4 increased the diameter of inhibition zones for the groups in both bacteria significantly (*p* < 0.0001). Tukey's post hoc test also showed that there was a significant difference between all groups and the 0% group in both bacteria that means even 0.5 percent of silica nanoparticles can lead to the significant antimicrobial action compared to the 0% group (Table 2).

The results also showed more sensitivity for *S. aureus* than *E. coli*. Balaure *et al.* also reported similar results for antimicrobial action of silica nanoparticles against *S. aureus* than *E. coli* [19]. Many studies have revealed that nanomaterials show better antimicrobial action against Gram-positive bacteria than against Gram-negative bacteria, due to the existence of lipoproteins and phospholipids in the cell wall of Gram-negative bacteria [20].

**Figure 5:**
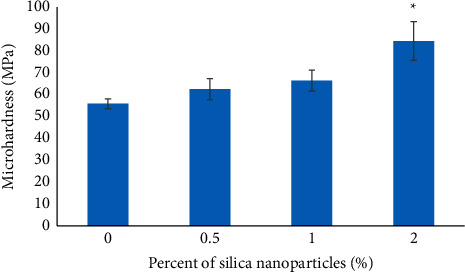
Results of hardness test. An asterisk represents a statistically significant difference (*p* < 0.05) between the groups (0.5%, 1%, and 2%) and 0% group.

The Vickers hardness test method also referred to the microhardness test is commonly applied for small parts, thin sections, or case depth studies [21]. According to the hardness results (Table 3 and Figure 5), adding silica nanoparticles to the dental stone type 4 increased the surface hardness significantly (*p* = 0.0057). Tukey's post hoc test (Table 4) also showed that the 0% and the 0.5% groups had significant differences with the 2% group (*p* = 0.0046 and *p* = 0.0205, respectively). It means that at least 2% silica nanoparticles are needed for significant increase in dental stone hardness compared to 0% of nanoparticles.

The reason for the increase in hardness with the addition of nanoparticles is due to the small size of the nanoparticles and the large surface area of the nanoparticles, which leads to a decrease in surface tension and an increase in the moisture content of the hemihydrates in dental stone. Therefore, the solubility of hemihydrate in water increases and the rate of crystallization occurs faster. As a result, the porosity of dental stone is reduced, which improves hardness [13, 22]. Also, the penetration of nanoparticles in the space between calcium phosphate crystals of dental stone and water absorption by them leads to the deposition of these nanoparticles in existing spaces [13]. Akkus *et al* suggested that the incorporation of nanoparticles to type III and type IV dental stones decreased the compressive strength [22]. De Cesero et al. found that the compressive strength for dental stone was not changed after the addition of silica nanoparticles [13].

## 5. Conclusion

Different treatments have been suggested to enhance the surface hardness of dental stones so far. A recent novelty in inorganic fillers has been the application of nanotechnology, with the main area of improving their properties. The results showed that adding silica nanoparticles to dental stone type 4 increased the surface hardness without any effect on physicochemical properties. Besides, the test groups demonstrated significant antimicrobial activity against *E. coli* and *S. aureus* compared to the control group (stone without nanoparticles).

## 6. Future Perspectives

By properly understanding the properties of dental nanomaterials, their specific strengths, limitations, and benefits will be better understood. Dental nanomaterials have the potential for the future, but there are currently few studies on their clinical applications. Clinical studies with the help of these nanomaterials in the field of dentistry are expected to increase in the future. It may result in improved life quality of patients. In future studies with the gypsum nanoparticle, we suggest the evaluation of setting expansion, setting time, and ability to reproduce details for experiments.

## Figures and Tables

**Table 1:** Inhibition zone measurements for the groups (0%, 0.5%, 1%, and 2%) and the positive controls against *S. aureus* and *E. coli*.

Samples	Inhibition zone (mm)	
	*E. coli*	*S. aureus*
0% silica NPs as negative control group	0	0
0.5% silica NPs	8.92 ± 0.09	9.10 ± 0.08
1% silica NPs	10.00 ± 0.25	11.36 ± 0.40
2% silica NPs	13.95 ± 0.10	15.26 ± 0.19
SS	377.2	377.2
DF	3	3
MS	125.7	125.7
F	32521	33091
*P*-value	<0.0001	<0.0001
Vancomycin (positive control group for gram-positive bacteria)	—	18.23 ± 0.2
Rifampicin (positive control group for gram-negative bacteria)	17.50 ± 0.09	—

**Table 2:** Results for Tukey's post hoc of antimicrobial test between the groups (0.5%, 1%, and 2%) and 0% group in both bacteria.

Statistical test	Groups	*p*-value *E. coli*	*p*-value *S. aureus*	q	DF
Tukey	0.5% group- 0% group	<0.0001	<0.0001	39.54	2
	1% group- 0% group	<0.0001	<0.0001	377.2	2
	2% group- 0% group	<0.0001	<0.0001	222.9	2
	0.5% group- 1% group	0.0490	<0.0001	95.53	2
	0.5% group- 2% group	<0.0001	<0.0001	695.7	2
	1% group- 2% group	<0.0001	<0.0001	623.0	2

**Table 3:** Results of hardness test for the groups (0%, 0.5%, 1%, and 2%).

Samples	Hardness (MPa)
0% silica NPs	55.85 ± 2.20
0.5% silica NPs	62.52 ± 4.83
1% silica NPs	66.42 ± 4.80
2% silica NPs	84.52 ± 8.91
SS	29589
DF	11
MS	2690
F	162.8
*p*-value	0.0057

**Table 4:** Results for Tukey's post hoc test hardness test between the groups (0.5%, 1%, and 2%) and 0% group.

Statistical test	Groups	*p*-value	Q	DF
Tukey	0.5% group- 0% group	**0.0046**	4.669	2
	1% group- 0% group	0.0600	3.400	2
	2% group- 0% group	**0.0205**	4.982	2
	0.5% group- 1% group	0.0790	3.124	2
	0.5% group- 2% group	0.0870	7.611	2
	1% group- 2% group	0.0990	5.290	2

## Data Availability

The raw data for this study can be shared at this time.
